# Delegating home visits in general practice: a realist review on the impact on GP workload and patient care

**DOI:** 10.3399/bjgp20X710153

**Published:** 2020-05-19

**Authors:** Ruth Abrams, Geoff Wong, Kamal R Mahtani, Stephanie Tierney, Anne-Marie Boylan, Nia Roberts, Sophie Park

**Affiliations:** Faculty of Health and Medical Sciences, School of Health Sciences, University of Surrey, Surrey.; Nuffield Department of Primary Care Health Sciences, University of Oxford, Oxford.; Nuffield Department of Primary Care Health Sciences, University of Oxford, Oxford.; Nuffield Department of Primary Care Health Sciences, University of Oxford, Oxford.; Nuffield Department of Primary Care Health Sciences, University of Oxford, Oxford.; Bodleian Health Care Libraries, University of Oxford, Oxford.; Department of Primary Care and Population Health, University College London, London.

**Keywords:** general practice, home visits, primary care, realist review, work delegation

## Abstract

**Background:**

UK general practice is being shaped by new ways of working. Traditional GP tasks are being delegated to other staff with the intention of reducing GPs’ workload and hospital admissions, and improving patients’ access to care. One such task is patient-requested home visits. However, it is unclear what impact delegated home visits may have, who might benefit, and under what circumstances.

**Aim:**

To explore how the process of delegating home visits works, for whom, and in what contexts.

**Design and setting:**

A review of secondary data on home visit delegation processes in UK primary care settings.

**Method:**

A realist approach was taken to reviewing data, which aims to provide causal explanations through the generation and articulation of contexts, mechanisms, and outcomes. A range of data has been used including news items, grey literature, and academic articles.

**Results:**

Data were synthesised from 70 documents. GPs may believe that delegating home visits is a risky option unless they have trust and experience with the wider multidisciplinary team. Internal systems such as technological infrastructure might help or hinder the delegation process. Healthcare professionals carrying out delegated home visits might benefit from being integrated into general practice but may feel that their clinical autonomy is limited by the delegation process. Patients report short-term satisfaction when visited by a healthcare professional other than a GP. The impact this has on long-term health outcomes and cost is less clear.

**Conclusion:**

The delegation of home visits may require a shift in patient expectation about who undertakes care. Professional expectations may also require a shift, having implications for the balance of staffing between primary and secondary care, and the training of healthcare professionals.

## INTRODUCTION

In the UK, home visits to patients are requested by or for patients if they are chronically or acutely housebound. Although some patients have informal or formal carers who are able to bring them to the surgery for an appointment, in many cases the patient’s health or social condition may prevent them from attending. Until very recently, home visits have been performed by a GP, traditionally after morning or afternoon surgeries. However, a vote by local medical committees (LMCs) in 2019 to remove home visits from core GP contracts suggests a need to re-evaluate the nature and organisation of home visits.^1^ The argument for this removal was cited as a lack of capacity among GPs to undertake home visits amid increasingly demanding workloads. However, the removal of home visits by GPs was not without opposition. Some GPs view home visits as a core part of general practice even though others see their removal as a positive step in reducing a potentially time-consuming task.^1^^–^^5^

Some practices are responding organically to increasing demands for timely patient appointments by delegating traditional GP-led home visits to another healthcare professional, such as an advanced nurse practitioner, locum GP, community paramedic, or emergency care clinician. Key assumptions associated with delegating home visits include that visiting a patient earlier in the day might reduce hospital admissions and that if the task is delegated to an alternative healthcare professional it will reduce a GP’s workload.^3^^,^^4^^,^^6^

The term ‘delegation’ is used specifically in recent NHS and policy documents.^3^^,^^4^^,^^7^ Delegation implies the breaking up and redirection of workflow from one worker to another, and has its roots in scientific management.^8^^,^^9^ The term delegation reflects the fact that the role and task of the GP are not entirely replaced, as seen in traditional substitution or task-shifting models.^10^ Currently, there is much variation in how the delegation of home visits is organised in England.^6^ The factors that make it successful are unclear and are likely to depend on a range of contexts. This review explores the ways in which delegated home visits impact on clinical workload and patient care, and aims to understand the roles and responsibilities of delegating home visits.

## METHOD

This review asks the following question: within the existing and available literature, what are the causal explanations for the ways in which home visit delegation contributes to patient care and clinical workload? This review explores how the process of delegating home visits works, for whom, and in what contexts. Home visit delegation was conceptualised as a complex intervention with outcomes that are context sensitive. In realist review methodology, exploring interventions (in this case delegation) as a multifaceted, social process enabled the authors to account for context, mechanisms, and outcomes associated with the evidence. By viewing delegation as a complex intervention, it is possible to interrogate the evidence to make visible the underlying assumptions about the intervention and explore the ways in which it may or may not work.^11^ A realist approach was used to carry out this review.^12^ Box 1 indicates the stages of this realist approach, which are in line with RAMESES guidelines.^13^ Further details about the realist review process undertaken in this study have been published elsewhere.^6^

**Table:** How this fits in

In November 2019, GPs in England voted to reduce home visits as part of their core contractual activities. However, the impact of this decision on both patient care and the wider workforce remains unclear. This realist review presents a number of causal explanations for why, whom, and when home visit delegation may or may not be useful to, and for, general practice. Findings suggest that a GP may feel that delegation is suitable if they have previously established a degree of professional trust with the healthcare professional (HCP) doing the home visit. This trust will facilitate the appropriate and safe sharing of information and follow-up deemed relevant to a particular case. GPs supporting home visit delegation should be mindful that this may not, in the long run, reduce their workload. This may be particularly pertinent if the patient has complex needs or if the HCP requires extensive input from the GP. However, the impact on patient health (and long-term outcomes) remains less clear.

**Box 1.** Realist review stages

Step 1: locating existing theories	Grey literature was sourced between April and June 2018. Keywords were used in academic databases, Google, and Google Scholar, including: primary care visiting services, home visiting services, early visiting services, acute visiting services, and general practice visiting services (see Supplementary Box S2 for details). Stakeholders including GPs, paramedics, and commissioners of this service were identified via professional networks of the team and Google searches. Perspectives, feedback, and advice were obtained. Written notes were taken in all conversations (*n*= 8) and used to inform the initial programme theory (see Supplementary Figure S1 for details).
Step 2: searching for evidence	Two searches were undertaken as part of this review and reflect the iterative nature of realist review searching.^13^^,^^14^ The first search aimed to identify empirical evidence (see Supplementary Box S3 for details of the main search strategy). The search strategy and the identification of extant literature was conducted with the support of an information specialist.
Screening	Documents were screened by one author using titles and abstracts, and then by full text; 10% random samples were reviewed independently by another author.
Additional searching	The second search was undertaken to aid the refinement of the processes associated with home visit delegation. Systematic literature reviews were located that focused specifically on delegation, for example, searching specifically for personnel delegation, task shifting/sharing, skill mix, and substitution.^14^ See Supplementary Box S4 for details of this search strategy.
Step 3: document selection	Full-text documents were selected for inclusion based on their ability to provide relevant data to the review. This included all documents used in an NHS setting or similar, as well as documents capable of identifying work delegation processes.
Step 4: data extraction	All included documents were coded in NVivo (version 12) (see Supplementary Tables S1–S4 for details of article characteristics, and Supplementary Tables S5 and S6 for details of the coding frameworks relating to each search).
Step 5: data synthesis	Working across and within coded data extracts, context–mechanism–outcome configurations (CMOCs) were developed as part of an iterative development of causal explanations (see Supplementary Boxes S5–S8 and Supplementary Figures S3–S6 for details of the CMOCs and their associated, partial programme theories. See Supplementary Table S7 for illustrative data reflective of each CMOC).
Step 6: refine programme theory	The final programme theory (see Supplementary Figure S2 for details) was taken back to the initial stakeholder group for refinement. A new stakeholder group of individuals, working more closely with those accepting delegated workloads to understand differences between GPs’ and other healthcare professionals’ perspectives, was also established.

Stakeholders were involved in the research as content experts, helping to establish the initial programme theory and refine the final programme theory (see Supplementary Figures S1 and S2 for details). This is a legitimate form of knowledge generation in realist reviewing.^14^ Patients were involved towards the end of the study to help refine the final programme theory. Findings from this review also helped to construct an interactive theatre performance to engage with the general public about the implications of the research findings in relation to practice.^15^

## RESULTS

**Figure 1.**
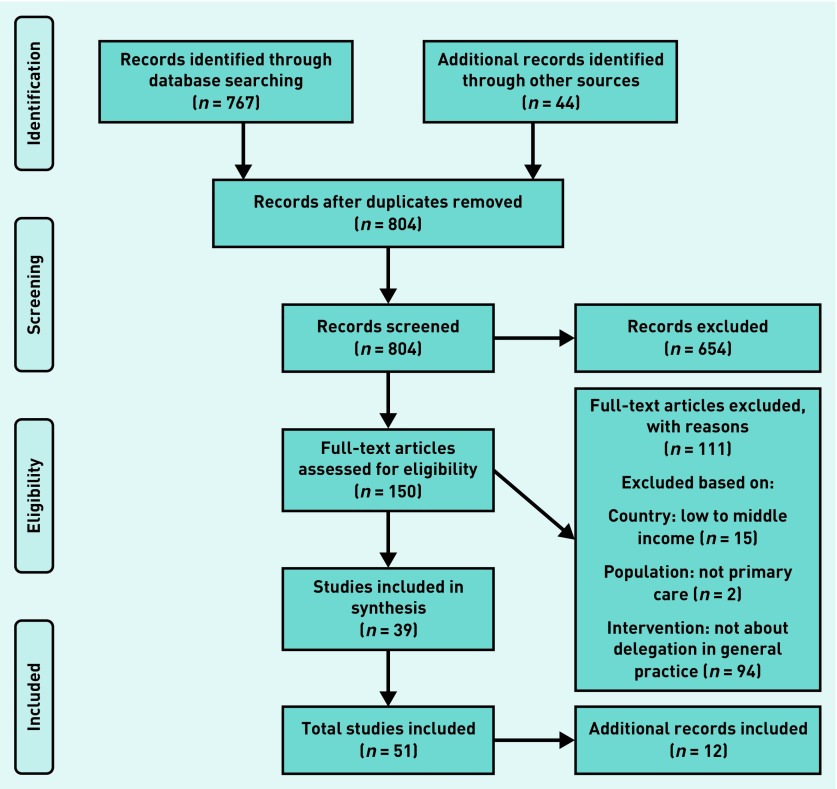
***PRISMA 1: primary searches carried out in June 2018.***

**Figure 2.**
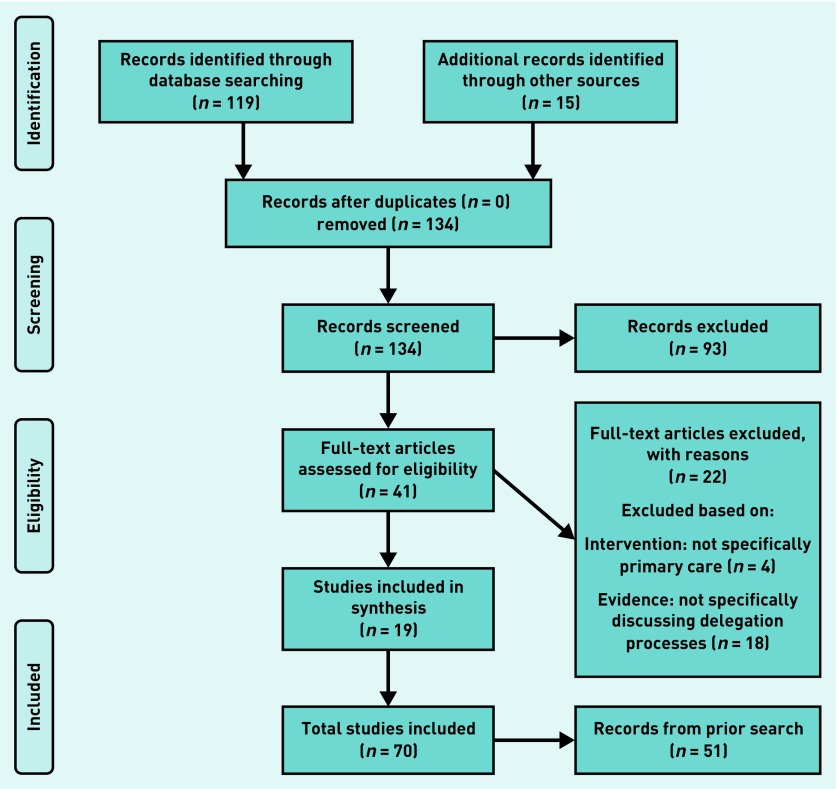
***PRISMA 2: purposive searches carried out in October 2018.***

In total, 70 documents were included in this review (Figures 1 and 2). Documents mainly referred to UK-based healthcare systems (*n* = 52). Academic literature (for example, Annis *et al* ^16^ and Edwards *et al* ^17^)comprised 53% (*n* = 37) of total documents included. Evaluations, policy documents, and thought pieces comprised 26% (*n* = 18) (for example, NHS Portsmouth Clinical Commissioning Group^18^), and media news items comprised 21% (*n* = 15) of documents referring to UK-based healthcare systems (for example, Duffin^19^ and Ford^20^).

The fact that half the documents in this review comprised localised, non-empirical or ancedotal forms of evidence has limited the depth of the explanations to some extent.^21^ However, these documents were deemed as trustworthy and capable of contributing to the coherence of the realist review because of their ability to contribute to answering the research questions.^22^ See Supplementary Boxes S1–S4 for the glossary and a full breakdown of the search strategies used, and Supplementary Tables S1–S4 for details of the documents included in the study.

The realist analysis is presented below. The findings are organised into three key issues: effective service design and system implementation; professional perspectives on home visit delegation; and factors affecting patient experience.

### Issue 1: Effective service design and system implementation

The way in which systems supporting the delegation of home visits are implemented in a GP practice has an impact on the interpersonal aspects of home visit delegation. For example, it might be beneficial to set up information technology systems so that the ability to share relevant information, such as a patient’s condition, the team member’s availability or capacity for home visits, and the skills available in the multidisciplinary network, are easy to access by those involved in triaging a home visit.^23^^,^^24^

Being clear about who the service is intended for might also help the member of staff delegating the visits to do so effectively.^24^^–^^26^ Enabling information to be shared and networks to be fostered is likely to provide a good foundation for effective coordination of the service. This may mean recruiting for a dedicated point of contact with knowledge of the available referral pathways. For example, this could be the patient’s ‘usual’ GP or another specifically recruited individual.^20^^,^^23^ This may be particularly important for a patient’s safety in instances where practices take referrals from other community services or outsource home visits.^23^^,^^26^

Having a system that is able to share any historical patient conditions appears crucial in determining the complexity of safely delegating a home visit (or not).^23^^,^^25^^,^^26^ A patient living with multiple illnesses may be too complex to delegate, but a patient with an acute need may not be.

Crucially, these systems can only be established once all relevant staff members are clear about the nature of the patient’s problem to be addressed and the role of the service.^25^^,^^26^

Systems also need to support a professional in a patient’s home, when the condition or illness might become more evident and require GP input.^3^^,^^23^^,^^26^ See Supplementary Box S5, Supplementary Figure S3, and Supplementary Table S7 (issue 1) for details.

### Issue 2: Professional perspectives on home visit delegation

#### The GP perspective

This issue explores the multitude of factors that are likely to influence whether a GP delegates a home visit (or not). For example, if they believe they cannot meet their professional demand as a result of an excessively high workload, they may feel compelled to delegate to another professional.^23^^,^^26^^–^^28^ This is most likely to occur in situations where a GP knows the healthcare professional or where they know information can flow between professionals appropriately to aid management of patient safety.^3^^,^^26^^,^^29^^,^^30^ Often this is a relationship developed over time, where cooperation and trust become established gradually.^30^^,^^31^

If a GP is not clear about the role and responsibilities of the healthcare professional, they are unlikely to delegate a task.^16^^,^^32^^–^^36^ This is particularly the case if they believe they can add more value to the patient by carrying out the visit themselves or if they do not trust the competence of the healthcare professional.^30^ Any doubt or confusion regarding the skills of the healthcare professional or the level of accountability may also prevent a GP from delegating the home visit.^27^^,^^37^^,^^38^ The disruption of professional boundaries in general practice may create feelings of unease, such as threat to a GP’s professional status, which may influence their openness to delegate.^38^^,^^39^ Ultimately, GPs might decide on a case-by-case basis, contingent on their established (or not) relationship with the healthcare professional,^27^^,^^40^^,^^41^ the patient, and their workload.^26^^,^^42^^–^^44^

Some evidence suggests that work from a delegated visit may return to GPs, increasing their workload with follow-up actions as an unintended consequence of the service.^26^ This finding is supported by wider evidence that suggests in some cases, when a healthcare professional sees a patient, they may generate additional work or duplicate work unnecessarily.^28^^,^^30^^,^^36^^,^^44^^,^^45^ See Supplementary Box S6, Supplementary Figure S4, and Supplementary Table S7 (issue 2) for details.

#### The healthcare professional perspective

Several documents positioned paramedics as capable members of the healthcare workforce, able to carry out delegated home visits because of their generalist skill mix, ability to prescribe (in some cases), and exposure to multidisciplinary teams and diverse environments.^46^^,^^47^ However, other documents suggest that home visits are not undertaken exclusively by paramedics and in some cases may be undertaken by nurse practitioners or locum GPs.^16^^,^^17^^,^^26^

Evidence found in the secondary searches indicates that, when undertaking delegated work, task variation,^48^ patient relationships,^38^ and clear identification of competencies^39^ are necessary ingredients to prevent confusion and frustration among staff. This presents a tension if individuals are employed exclusively to undertake delegated home visits.

Autonomy over tasks is a key factor in providing job satisfaction when accepting delegated work.^48^ However, when receiving delegated work, healthcare professionals may not be able to, are not required to, or do not have the necessary responsibility that enables them to exercise their full clinical judgement. The evidence suggests that healthcare professionals appear frustrated when tasks they can normally undertake in their ‘typical’ environment become restricted because of different liabilities or risk management strategies.^31^^,^^39^^,^^40^ The ability for healthcare professionals to receive professional feedback also contributes to their feelings about receiving and undertaking delegated work tasks.^28^^,^^31^ Therefore, isolation may be an issue for those not fully integrated into their care environment.^38^

The quality of the relationship between GPs and other healthcare professionals appears equally significant. From the perspective of the healthcare professional, conflict may arise if they believe that GPs are working against them.^31^

The degree to which healthcare professionals are accepted into the fold of general practice appears to influence both how able they feel to carry out the delegated task and how respected they feel in the environment in which they are working.^31^^,^^34^ For example, responsiveness to care queries, the sharing of documentation, and overall communication between GPs and healthcare professionals are all interactions that can hinder or help the formation of respect between professionals, as is the willingness of GPs to view healthcare professionals as knowledgeable.^30^ This is evidence previously documented in the establishment of relationships between GPs and nurse practitioners during work delegation.^29^^–^^31^ See Supplementary Box S7, Supplementary Figure S5, and Supplementary Table S7 (issue 2) for details.

### Issue 3: Factors affecting patient experience

For patients experiencing a delegated home visit, data suggest that, when they are seen quickly, by a caring and competent healthcare professional, their (short-term) expectations are met. Specifically, patients value having their health concern taken seriously with a speedy response, having their minds put at ease with explanations, being given advice on their condition, and receiving more time and attention from a qualified professional in an unpressured way.^17^^,^^26^^,^^49^

In cases where patients are seen earlier in the day, they appreciate having more options than being referred straight to hospital.^26^ Evidence found in secondary searches indicates that healthcare professionals spend longer with patients, educating them about self-care, management, and advice.^50^^,^^51^ Data also suggest that patients often view other healthcare professionals as being as competent as GPs.^38^^,^^39^^,^^52^

Some patients, however, do prefer to see their own GP because of familiarity.^23^ This might be dependent on the patient’s condition. In instances where a condition is chronic, patients do not feel comfortable seeing another practitioner.^23^^,^^38^^,^^44^ This might also be because of a certain wariness and cautiousness about the role of the healthcare professional.^38^ See supplementary Box S8, Supplementary Figure S6, and Supplementary Table S7 (issue 3) for details.

## DISCUSSION

### Summary

This review sought to understand and explain delegation of home visits in general practice. Although the service is still emerging across the country, this synthesis includes a number of considerations to support improved implementation and impact. It also highlights potential limitations. For a GP’s workload to be reduced, their involvement in the delegation process and any resulting deferral of work needs to be minimal. Yet GPs are highly attuned to managing risk in primary care. This review suggests that GPs may find themselves asking whether it is safe to delegate and whether any value (such as saving time, reducing workload, or earlier assessment) is added as a result of delegating. Balancing acute and complex patient needs across delegated and GP roles is important to avoid worsening a sense of GP workload.

The integration of a wider workforce, for example, with paramedics, locum GPs, and nurse practitioners, to undertake delegated home visits may be beneficial to patients. However, the findings of this study suggest that professionals entering into the workforce with previous experience and qualifications may become frustrated if their clinical autonomy is limited or undermined by the delegation process. The findings also suggest that patient satisfaction is often high when seen by another healthcare practitioner, such as a nurse, but the long-term implications this has on their health are less clear. Whether home visit delegation is useful to long-term patient care and hospital admission reductions is not known.

Careful consideration is also required to ensure delegation does not increase healthcare access inequities. Sustainable organisation of care through delegation requires analysis of the positives and negatives to staff, patients, and at a societal level, as well as an understanding of for whom, when, and in what circumstances a delegated visit is desirable.

### Strengths and limitations

Evidence for home visit delegation is still emerging. The findings of this study are based on interventions that are at the forefront of primary care development (see Nabhani-Gebara *et al* ^53^ and Turner *et al* ^54^). The strength of using a realist approach is the ability to make use of a range of data that might not have been considered in a conventional systematic review. Specifically, many of the context–mechanism–outcome configurations (CMOCs) in the study were built from grey literature, which provided contextual information. Although this helped to build a picture of home visit delegation through the study’s programme theory, it may mean that replication of the searches used will be a challenge^55^ and have implications for the trustworthiness of some of the CMOCs. As more empirical data become available on these services, further refinement, confirmation, and refutation of the study’s findings are anticipated.

### Comparison with existing literature

Delegation relies on clear role boundaries and clear patient conditions. However, there is limited scope in general practice for patient problems to be ‘pre-defined’.^4^ Often, until a patient has been assessed, their health issue is not clear and this may produce confusion regarding responsibility. Lower healthcare expenditure is associated with a GP’s ability to carry out gatekeeping because of their ability to refer appropriately and in a timely manner.^56^ The current review demonstrates that delegation in general practice may have a direct impact on a GP’s ability to carry out gatekeeping.

Existing literature on the role of healthcare professionals in primary care suggests that professionals who have control over their clinical practice are more likely to enjoy their work and experience a greater sense of accomplishment.^57^ Limits placed on a professional group in terms of clinical autonomy may lead to a greater likelihood of resignation.^57^^,^^58^ In this new and expanding multidisciplinary environment, the current review suggests a need to consider the retention, training, and support for this evolving workforce.

### Implications for research and practice

With the release of the *NHS Long Term Plan*,^3^ there is a clear shift towards using a more multidisciplinary primary care workforce. However, introducing a broader workforce into primary care and delegating tasks such as home visits may have wider-reaching implications for the balance of staff between primary and secondary care, as well as the acute services. There may also be implications for staff training and workforce retention.

Although the cost-effectiveness of delegating workloads remains a contentious issue,^59^^,^^60^ service improvement has been a consistent finding in regards to using the skills of the wider healthcare workforce.^32^ Resource utilisation,^40^ societal cost,^44^ the ability of a service to enhance long-term care provision,^38^^,^^39^ and professional development are issues that require further consideration.

Effective service evaluations involving data comparisons between GPs’ and healthcare professionals’ time and workloads need to be built into pilot programmes for fair comparisons to be made regarding care quality outcomes. Although some examples of this are happening in practice,^18^ a lack of focused collaboration and planned evaluations may help account for inequalities across disparate areas of the country.

Box 2 summarises the practice implications of the study’s findings at the organisational, professional, and policy levels, and highlights potential pitfalls to avoid. These are based on the current available data on home visit delegation. Box 2 also provides information for clinicians to make judgements (based on the findings of the current review) of what can currently be addressed when delegating home visits. Not all the principles and watch points will be relevant to all services that seek to delegate home visits, but Box 2’s contents may be useful to support decision making or service improvements.

**Box 2.** Summary of practice implications

**Implementation level**	**Principles to encourage**	**Watch points**
Organisational level	Information sharing of staff availability, staff skill set, and patient medical histories among healthcare professionals. Appropriate communication of staff roles to patients to encourage patient receptiveness. Integration of all staff members into practice.	Unnecessary limits/restrictions placed on staff with clinical skills and the ability to make clinical judgements. Continuity of care for patients with complex needs. Organisational cultures that do not foster environments for interprofessional trust and collaboration.
Professional level	Interprofessional dialogue and communication. Preparedness, autonomy, and respectful relationships. Establishment and management of patient expectations.	Ineffective feedback loops and deferred workloads. Staff frustration, despondency, and professional isolation. Patient reluctance to see a different healthcare professional.
Policy level	Sustainable, long-term management of delegation processes. Enhancement of opportunities for clinical supervision, training, and preparation.	Evaluation of long-term patient health outcomes and cost implications. Balance of staff between primary and secondary care/staff retention.

Delegation of home visits demands collaboration, therefore practices and individuals need to establish trust, respect, communication, and interaction between professionals for delegation to work. At a structural level, implementation of adequate systems that facilitate communication and outline responsibilities needs to be considered. This needs to be done early on in the implementation of the new service. Practices and training providers alike need to begin to raise awareness at an interprofessional level about the responsibilities and risk involved in work delegation, and the impact this has on patient expectation and workflow.

The present study findings and causal explanations might help with future implementation of home visit delegation, with some potential lessons for other related delegation of work in primary care.

Future research may begin to focus on the limited data regarding cost-effectiveness and patient health outcomes relating to home visit delegation.
